# *In Vitro* Efficacy of Gentamicin Alone and in Combination with Ceftriaxone, Ertapenem, and Azithromycin against Multidrug-Resistant Neisseria gonorrhoeae

**DOI:** 10.1128/Spectrum.00181-21

**Published:** 2021-10-20

**Authors:** Xuechun Li, Wenjing Le, Xiangdi Lou, Biwei Wang, Caroline A. Genco, Peter A. Rice, Xiaohong Su

**Affiliations:** a Institute of Dermatology, Chinese Academy of Medical Sciences and Peking Union Medical College, Nanjing, China; b Department of Immunology, School of Medicine, Tufts University, Boston, Massachusetts, USA; c Division of Infectious Diseases and Immunology, Department of Medicine, University of Massachusetts Chan Medical School, Worcester, Massachusetts, USA; University of Cincinnati

**Keywords:** *Neisseria gonorrhoeae*, multidrug resistance, combination, gentamicin

## Abstract

This study was conducted to determine the *in vitro* activities of gentamicin alone and in combination with ceftriaxone, ertapenem, and azithromycin against multidrug-resistant (MDR) Neisseria gonorrhoeae isolates. A total of 407 clinical isolates from Nanjing, China, obtained in 2016 to 2017, had MICs determined for gentamicin using the agar dilution method. MDR status was ascribed to 97 strains that displayed decreased susceptibility or resistance to extended-spectrum cephalosporins (ESCs) (ceftriaxone [MIC, ≥0.125 mg/liter] and cefixime [MIC, ≥0.25 mg/liter]), plus resistance to at least two of the following antimicrobials: penicillin (MIC, ≥2 mg/liter), ciprofloxacin (MIC, ≥1 mg/liter), and azithromycin (MIC, ≥1 mg/liter). MDR strains underwent MIC determinations for antimicrobial combinations using the antimicrobial gradient epsilometer test (Etest). Results that ranged from synergy to antagonism were interpreted using the fractional inhibitory concentration (FICI). All 407 gonococcal isolates were susceptible to gentamicin; MICs ranged from 2 mg/liter to 16 mg/liter. Synergy was demonstrated in 16.5% (16/97), 27.8% (27/97), and 8.2% (8/97) of MDR strains when gentamicin was combined with ceftriaxone (geometric mean [GM] FICI, 0.747), ertapenem (GM FICI, 0.662), and azithromycin (GM FICI, 1.021), respectively. No antimicrobial antagonism was observed with any combination tested against MDR strains; overall, antimicrobial combinations were indifferent. The GM MICs of gentamicin were reduced by 2.63-, 3.80-, and 1.98-fold when tested in combination with ceftriaxone, ertapenem, and azithromycin, respectively. The GM MICs of the three additional antimicrobials individually were reduced by 3-, 2.57-, and 1.98-fold, respectively, when each was tested in combination with gentamicin. Gentamicin alone was effective *in vitro* against N. gonorrhoeae, including MDR isolates. Combination testing of MDR strains showed lower MICs against gentamicin and each of three antimicrobials (ceftriaxone, ertapenem, and azithromycin) when used in combination.

**IMPORTANCE** Antimicrobial-resistant Neisseria gonorrhoeae is a major global public health concern. New treatment options are urgently needed to successfully treat multidrug-resistant (MDR) Neisseria gonorrhoeae infections. This study showed that gentamicin maintained excellent *in vitro* susceptibility against clinical gonococcal isolates collected in 2016 and 2017, including MDR isolates. Combinations of gentamicin plus ertapenem, ceftriaxone, and azithromycin produced synergistic effects against certain MDR isolates. No antagonism was observed in any of the antimicrobial combinations, which may prove useful to guide clinical testing of combination therapies.

## INTRODUCTION

Gonorrhea, caused by Neisseria gonorrhoeae, is currently the second most common bacterial sexually transmitted infection (STI) worldwide and, accordingly, is a major public health problem globally. In 2016, the World Health Organization (WHO) estimated 87 million new cases of gonorrhea worldwide in adults age 15 to 49 (1). Common features of gonococcal infection include cervicitis and urethritis. If untreated, infection can spread to the upper genital tract and cause pelvic inflammatory disease in women and epididymitis in men. Long-term complications in women include chronic pelvic pain and infertility. Disseminated gonococcal infection (DGI) occurs occasionally in adults. Neonates born to infected mothers can develop ophthalmia neonatorum and, rarely, DGI. In the preantibiotic era endocarditis and meningitis sometimes resulted from gonococcal bacteremia but are rarely seen today. Concomitant gonococcal and HIV infections also increase the risk of HIV transmission (2).

The emergence of antibiotic resistance among N. gonorrhoeae is a global public health threat. N. gonorrhoeae has developed resistance to antimicrobials that have been used historically for treatment, including sulfonamides, penicillins, tetracyclines, and fluoroquinolones, leading to the emergence of multidrug-resistant (MDR) isolates, which are difficult to treat (3). Currently, as a strategy for preventing extended-spectrum cephalosporin (ESC) resistance and to treat possible coinfection with Chlamydia trachomatis, the WHO recommends dual antimicrobial therapy with an ESC, either ceftriaxone (250 mg intramuscularly) or cefixime (400 mg orally), plus azithromycin (1 g orally), as a first-line treatment of uncomplicated gonorrhea (4). The increased prevalence of azithromycin resistance globally prompted a revision of prior recommendations in the United Kingdom (5) in 2018 and the United States (6) in 2020 from dual therapy with ceftriaxone and azithromycin to monotherapy with higher doses of ceftriaxone—1 g (United Kingdom) and 500 mg (United States). Unfortunately, resistance to ESCs (7–9), the last remaining option for empirical first-line monotherapy, threatens future use of this class of antimicrobials. Treatment failures with both mono- and dual-therapy (including azithromycin) have been reported in recent years (10, 11).

Treatment options for gonorrhea, including infections caused by MDR organisms, are diminishing; there is an urgent need to explore new or repurposed antimicrobial agents and/or therapeutic strategies. Gentamicin, an aminoglycoside antibiotic that inhibits protein synthesis by binding to the 30S ribosomal subunit, has been used as a first-line therapy for the treatment of gonorrhea in several countries, including Malawi, where it has been used officially for nearly 30 years (12). Numerous *in vitro* susceptibility studies have shown that gentamicin is active against N. gonorrhoeae, including MDR strains and strains with decreased susceptibility to currently recommended ESCs (13–16). A randomized noninferiority trial showed that the efficacy of gentamicin (91% effective) for the treatment of gonorrhea was inferior to the efficacy of ceftriaxone (98% effective) (both combined with azithromycin), suggesting that gentamicin is not appropriate as the first-line treatment for gonorrhea but remains potentially useful for patients who are allergic or intolerant to ceftriaxone or harbor an MDR isolate (17). Little is known about the *in vitro* susceptibility of gentamicin in isolates from China, where this antimicrobial has not been used to treat gonorrhea. Use of antimicrobial combinations is a therapeutic strategy intended to increase efficacy and slow the development of resistance (18). Antimicrobial combinations may prove useful to successfully manage MDR N. gonorrhoeae infections, and they are included in current WHO and CDC guidelines (4, 6).

Initially, we evaluated the gentamicin susceptibility of gonococcal strains isolated from 2016 to 2017 in Nanjing, China. Second, we carried out studies with antimicrobial combinations that included gentamicin to evaluate possible *in vitro* enhancement of gentamicin activity against MDR strains when tested in combination with either ceftriaxone (an ESC), ertapenem (a carbapenem), or azithromycin (a macrolide). Third, we determined the MICs of each of the three antimicrobials individually when tested in combination with gentamicin.

## RESULTS

**FIG 1:**
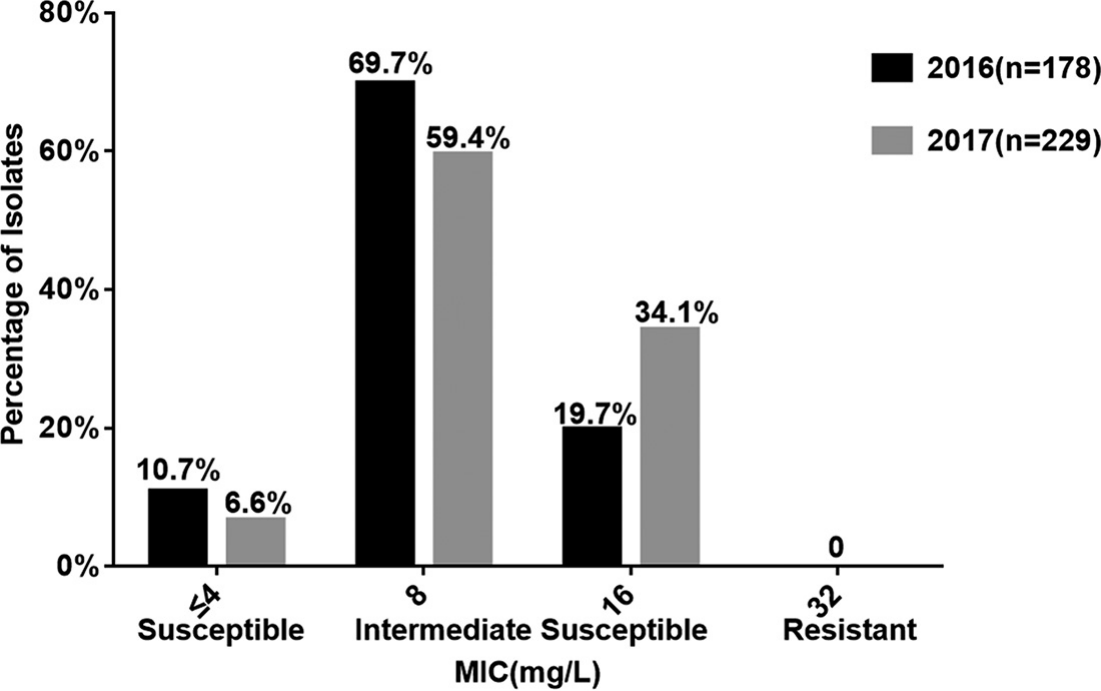
Distributions of MICs of gentamicin against N. gonorrhoeae, including 97 MDR isolates in 2016 (*n* = 178) and 2017 (*n* = 229).

All 407 N. gonorrhoeae strains were susceptible to gentamicin by agar dilution; MICs ranged from 2 mg/liter to 16 mg/liter; the MIC_50_ was 8 mg/liter and the MIC_90_ was 16 mg/liter. Among the 407 isolates, 34 (8.4%) were susceptible (MIC, ≤4 mg/liter), 373 (91.6%) were intermediately susceptible (MIC range, 8 to 16 mg/liter), and none was resistant (MIC, ≥32 mg/liter). Among the 34 fully susceptible isolates, 6 (17.6%) had decreased susceptibility to ESCs, i.e., ceftriaxone or cefixime or both, and 1 (2.9%) was fully resistant to ceftriaxone (MIC, 1 mg/liter) and cefixime (MIC, 2 mg/liter). There was no change in intermediate susceptibility to gentamicin of isolates from 2016 to 2017 (89.3% versus 93.4%; χ^2^ = 2.225, *P* = 0.136) (Fig. 1).

The distribution of antimicrobial susceptibility patterns for the 97 MDR strains selected for antimicrobial combination testing showed 6 unique patterns of susceptibility (see Table S1 in the supplemental material). Among MDR strains selected for antimicrobial combination testing, 93 (95.9%) strains had decreased susceptibility to ceftriaxone or cefixime or both plus resistance to ciprofloxacin and penicillin, and 4 (4.1%) strains had decreased susceptibility to ceftriaxone or cefixime or both plus resistance to ciprofloxacin, penicillin, and azithromycin. Resistance to gentamicin in MDR isolates was not detected by either the agar dilution or the Etest method. Agreement of MICs between agar dilution and Etest among MDR isolates is summarized in Table S2. The categorical agreement (CA) rate of MICs between the two methods was 79.4%, and the essential agreement (EA) rate (≤2-fold different) was 93.8%. Etest always resulted in 1 to 2 dilutions lower MIC values than agar dilution. Gentamicin agar dilution versus Etest MIC results (mg/liter) were as follows: MIC_50_, 8 versus 6; MIC_90_, 16 versus 8; geometric mean (GM) MIC, 11.3 versus 5.84. Additionally, there was a discrepancy in the full susceptibility category (29.9% fully susceptible by Etest versus 9.3% by agar dilution; χ^2^ = 13.090, *P* < 0.001).

The three antimicrobial combinations used to test each of the 97 strains were examined for effects that were classified as synergistic, indifferent, or antagonistic (summarized in Table 1). For example, the gentamicin GM MIC, when tested alone, was 5.840 mg/liter; when combined with ceftriaxone, the GM MIC was reduced to 2.217 mg/liter (2.63-fold reduction, *P* < 0.001) (Table 2). Together with ceftriaxone, gentamicin exhibited synergy against 16.5% (16/97) of MDR strains; overall, the combination was indifferent (GM FICI, 0.747). When tested alone, MICs of ceftriaxone ranged from 0.016 to 0.75 mg/liter; in combination with gentamicin, the GM MIC against ceftriaxone decreased from 0.078 to 0.026 mg/liter (3-fold reduction, *P* < 0.001).

**TABLE 1:** Synergy test results for combinations of gentamicin plus ceftriaxone, ertapenem, and azithromycin against 97 MDR N. gonorrhoeae isolates^*a*^

Effect	Data for antimicrobial combinations		
	GEN + CRO	GEN + ETP	GEN + AZM
Synergistic [*n* (%)]	16 (16.5)	27 (27.8)	8 (8.2)
Indifferent [*n* (%)]	81 (83.5)	70 (72.2)	89 (91.8)
Antagonistic [*n* (%)]	0	0	0
FICI (geometric mean)	0.747	0.662	1.021
Classification overall	Indifferent	Indifferent	Indifferent

a *n*, number; GEN, gentamicin; CRO, ceftriaxone; ETP, ertapenem; AZM, azithromycin; FICI, fractional inhibitory concentration.

**TABLE 2:** Etest MICs of the indicated antibiotics alone and in combination against 97 MDR N. gonorrhoeae isolates^*a*^

Antimicrobial combination	GM MIC (mg/liter) (range)
GEN + CRO	
GEN	
Alone	5.840 (2–12)
Combination	2.217 (0.38–6)
CRO	
Alone	0.078 (0.016–0.75)
Combination	0.026 (0.004–0.25)
GEN +ETP	
GEN	
Alone	5.840 (2–12)
Combination	1.536 (0.25–6)
ETP	
Alone	0.018 (0.006–0.064)
Combination	0.007 (0.002–0.047)
GEN +AZM	
GEN	
Alone	5.840 (2–12)
Combination	2.949 (1–8)
AZM	
Alone	0.347 (0.047–8)
Combination	0.175 (0.023–2)

a GEN, gentamicin; CRO, ceftriaxone; ETP, ertapenem; AZM, azithromycin; GM, geometric mean.

The GM MIC of gentamicin when combined with ertapenem decreased to 1.536 mg/liter (3.80-fold reduction, *P* < 0.001) (Table 2). Gentamicin together with ertapenem was the most synergistic combination, displaying synergy against 27.8% (27/97) of MDR gonococcal isolates; overall, this combination was indifferent (GM FICI, 0.662). Ertapenem MICs of 97 MDR isolates, when tested alone, ranged from 0.006 to 0.064 mg/liter (GM MIC, 0.018 mg/liter); when ertapenem was combined with gentamicin, the GM MIC decreased to 0.007 mg/liter (2.57-fold reduction, *P* < 0.001).

The GM MIC of gentamicin when combined with azithromycin decreased to 2.949 mg/liter (1.98-fold reduction, *P* < 0.001) (Table 2). Together with azithromycin, gentamicin exhibited synergy against 8.2% (8/97) of MDR strains; overall, the combination was indifferent (GM FICI, 1.021). When tested alone, azithromycin MICs ranged from 0.047 to 8 mg/liter; in combination with gentamicin, the GM MIC against azithromycin decreased from 0.347 to 0.175 mg/liter (1.98-fold reduction, *P* < 0.001).

## DISCUSSION

Our study provides data on gentamicin susceptibility against N. gonorrhoeae isolated in Nanjing (Jiangsu Province), China. On agar dilution testing, most strains displayed intermediate susceptibility to gentamicin (MIC, 8 to 16 mg/liter), similar to a study that examined gonococcal isolates from seven hospitals in a neighboring eastern Chinese province; in that study 97.8% (493/504) of strains possessed gentamicin MICs of 8 to 16 mg/liter (19). European and U.S. studies have reported 82.7% (13) and 73% (16) intermediate susceptibility, respectively, of N. gonorrhoeae isolates to gentamicin. A recent report of gentamicin susceptibility of N. gonorrhoeae in which 86.0% of 470 isolates were fully susceptible (MICs, ≤4 mg/liter) examined isolates from seven geographically distributed Chinese provinces as part of the China Gonococcal Resistance Surveillance Programme (China-GRSP) (20), similar to an Indian study where 90.7% of isolates were reported as fully susceptible (15). Our study compared gentamicin MICs using agar dilution and Etest methods for 97 multidrug-resistant (MDR) N. gonorrhoeae isolates. Similar to previous studies (13, 21), we found that over 90% of gentamicin MICs determined by agar dilution and Etest were ≤2-fold different; typically, Etest resulted in lower MICs and identified a larger proportion of fully susceptible isolates. In particular, all MDR isolates were fully or intermediately susceptible to gentamicin.

Synergistic or additive effects of combining antimicrobials for treatment may slow the development of antimicrobial resistance of N. gonorrhoeae (22). We assessed the *in vitro* activity of gentamicin in combination with 3 antibiotics. Determining synergy has several challenges. Several test methods are available to evaluate the synergistic effects of antimicrobial combinations; however, they are not well standardized. We chose Etest because it is practical and correlates well with agar dilution, time-kill curves, and checkerboard testing in demonstrating synergy for two-drug combinations (23–25). Nonetheless, FICI interpretation depends on the criteria used. FICI values between 0.5 and 1 have been interpreted as additive in Indian (26) and Japanese (27) studies, differing from our criteria, which classifies FICI values in this range as indifferent.

Ceftriaxone, in higher doses, is now recommended as single therapy by the United States and the United Kingdom for treatment of uncomplicated gonorrhea (5, 6). Our study showed that the combination of ceftriaxone and gentamicin exhibited an indifferent effect overall in >80% of MDR strains (<20% synergy). In the Indian study by Singh et al. (26), 14.7% synergy and 6.3% antagonism were reported for this combination against 95 N. gonorrhoeae strains, including 79 MDR and 1 extensively drug-resistant (XDR) strain. In a Canadian study, a mean 50% FICI (FICI_50_) value of 1.2 (range, 0.8 to 2.0) was shown for nine reference strains of N. gonorrhoeae (WHO F, G, K, L, M, N, O, and P and ATCC 49226) with this combination (28). A U.S. study reported a mean FICI of 1.25 (range, 0.73 to 2) using gonococcal isolates that displayed different cefixime MICs (24). No synergistic/antagonistic effect (resulting in 100% indifference) was observed in either study (24, 28).

We chose ertapenem as a candidate for *in vitro* synergy testing because its mechanism of action differs from that of gentamicin and it has been used to treat infection with combined high-level azithromycin- and ceftriaxone-resistant N. gonorrhoeae (11). Ertapenem has demonstrated an advantage over ceftriaxone for MDR or ceftriaxone-resistant isolates and has also been suggested for possible use in a dual antimicrobial regimen (29). We showed that gentamicin plus ertapenem in combination resulted in synergistic and indifferent effects, with no antagonism demonstrated in any MDR strain. In the study by Singh et al. (26), this combination displayed either synergy (31.6%) or indifference (68.4%) in 100% of strains; no antagonism was seen.

Gentamicin in combination with azithromycin is currently recommended by the WHO as an option for retreatment when dual therapy fails (4). Also, it is proposed as an alternative CDC recommendation when a higher dose ceftriaxone therapy can not be used (6). In our studies, this combination demonstrated synergy in fewer MDR isolates (<10%) than combinations with either ceftriaxone or ertapenem and exhibited the highest FICI value of the three combinations tested. Similar to our results, Sood et al. (30) demonstrated synergistic effects in 22.9% of isolates displaying different ceftriaxone MICs and no antagonism for this combination. Studies from the United Kingdom (31) and Japan (27) have reported indifference with a mean FICI of 1.7 and 0.83, respectively, among isolates with different cefixime/ceftriaxone MICs. The study by Singh et al. (26) differed from these results, with 6.3% of strains exhibiting antagonism when this combination was used.

No synergistic/antagonistic effect (resulting in 100% indifference) was observed in studies from Canada (28), Japan (27), the United States (24), and the United Kingdom (31). None of these studies incorporated isolates with multidrug resistance. However, a certain proportion of synergistic effects was observed in the two Indian studies (26, 30). Antagonism was also observed in combinations of gentamicin with ceftriaxone and azithromycin in the study by Singh et al. (26). Sood et al. (30) used the same Etest that we used. In contrast, Singh et al. (26) incubated the Etest strip of the first antimicrobial for 1 h and then replaced it with the Etest strip of the second antimicrobial at the same location and looked for synergism. A mild degree of antagonism may have been missed in our tests when the zone of inhibition ran under the strips where they crossed and therefore was unreadable and interpreted as indifference (23). A summary of results from these previous studies is shown in Table S3.

From a pharmacokinetic perspective, all antimicrobials in the 3 combinations in our study result in peak levels of drug during the first 3 h after administration (32–35). However, azithromycin has a longer half-life than gentamicin (approximately 68 and 2 h, respectively) (32, 35). Gentamicin in combination with azithromycin also produced the lowest synergistic effects among the 3 combinations in our study, so it may not be optimal for clinical use where synergy would not be prolonged.

In conclusion, resistance to gentamicin was not observed in gonococcal isolates examined in this study, including MDR isolates. Antimicrobial combinations of gentamicin plus ertapenem, ceftriaxone, and azithromycin showed no antagonistic effects; enhanced efficacy of individual antimicrobials in the presence of other antimicrobials was also demonstrated. Gentamicin has been effective in treating gonorrhea generally (36) and might also be an effective treatment option for MDR strains in combination with ertapenem, ceftriaxone, or azithromycin. Further studies to correlate *in vitro* results with clinical outcomes and establish clinical breakpoint criteria are warranted.

## MATERIALS AND METHODS

### Bacterial strains.

A total of 407 gonococcal isolates were recovered from men with symptomatic urethritis (urethral discharge and/or dysuria) attending the sexually transmitted disease (STD) clinic at the Institute of Dermatology, Chinese Academy of Medical Sciences, in Nanjing, China, from January 2016 to December 2017. Urethral specimens were collected with cotton swabs and immediately streaked onto modified Thayer-Martin medium (Zhuhai DL Biotech Co. Ltd.) and cultured in candle jars at 36°C for 24 to 48 h. N. gonorrhoeae was identified by colonial morphology, Gram’s stain, and oxidase testing, which are sufficient to identify N. gonorrhoeae colonies isolated on selective medium, particularly for samples from the urethral tracts of symptomatic men (37, 38). Isolates were subcultured onto GC chocolate agar base (Difco, Detroit, MI) supplemented with 1% IsovitaleX (Oxoid, USA); pure cultures were swabbed, suspended in tryptone-based soy broth, and frozen (−80°C) until being used for antimicrobial testing.

### Antimicrobial susceptibility testing.

The MICs of the 407 isolates were determined using the agar dilution method for gentamicin, penicillin, tetracycline, ciprofloxacin, azithromycin, spectinomycin, cefixime, and ceftriaxone, used singly, according to the Clinical and Laboratory Standards Institute (CLSI) guidelines (39). N. gonorrhoeae ATCC 49226 and WHO reference strains F, G, L, O, and P were used as quality control strains in susceptibility tests. Although formal susceptibility criteria for gentamicin have not been established by the CLSI (39) or the European Committee on Antimicrobial Susceptibility Testing (EUCAST) (40), criteria based on previous MIC comparisons and clinical cure data have characterized MICs of ≤4 mg/liter as fully susceptible, 8 to 16 mg/liter as intermediately susceptible, and ≥32 mg/liter as resistant (16). Resistance to azithromycin (MIC, ≥1 mg/liter) was determined using EUCAST criteria (40). Susceptibilities to other antibiotics were assessed based on CLSI standards (39). Decreased susceptibility to cephalosporins was determined according to WHO standards (41). Based on criteria proposed by Tapsall et al. in 2009 (42), MDR isolates were defined as those resistant or with decreased susceptibility to one or more widely used antimicrobials (ceftriaxone and cefixime) and resistant to two or more antimicrobials which are used less frequently (penicillin, ciprofloxacin and azithromycin).

### Synergy testing and interpretation.

**FIG 2:**
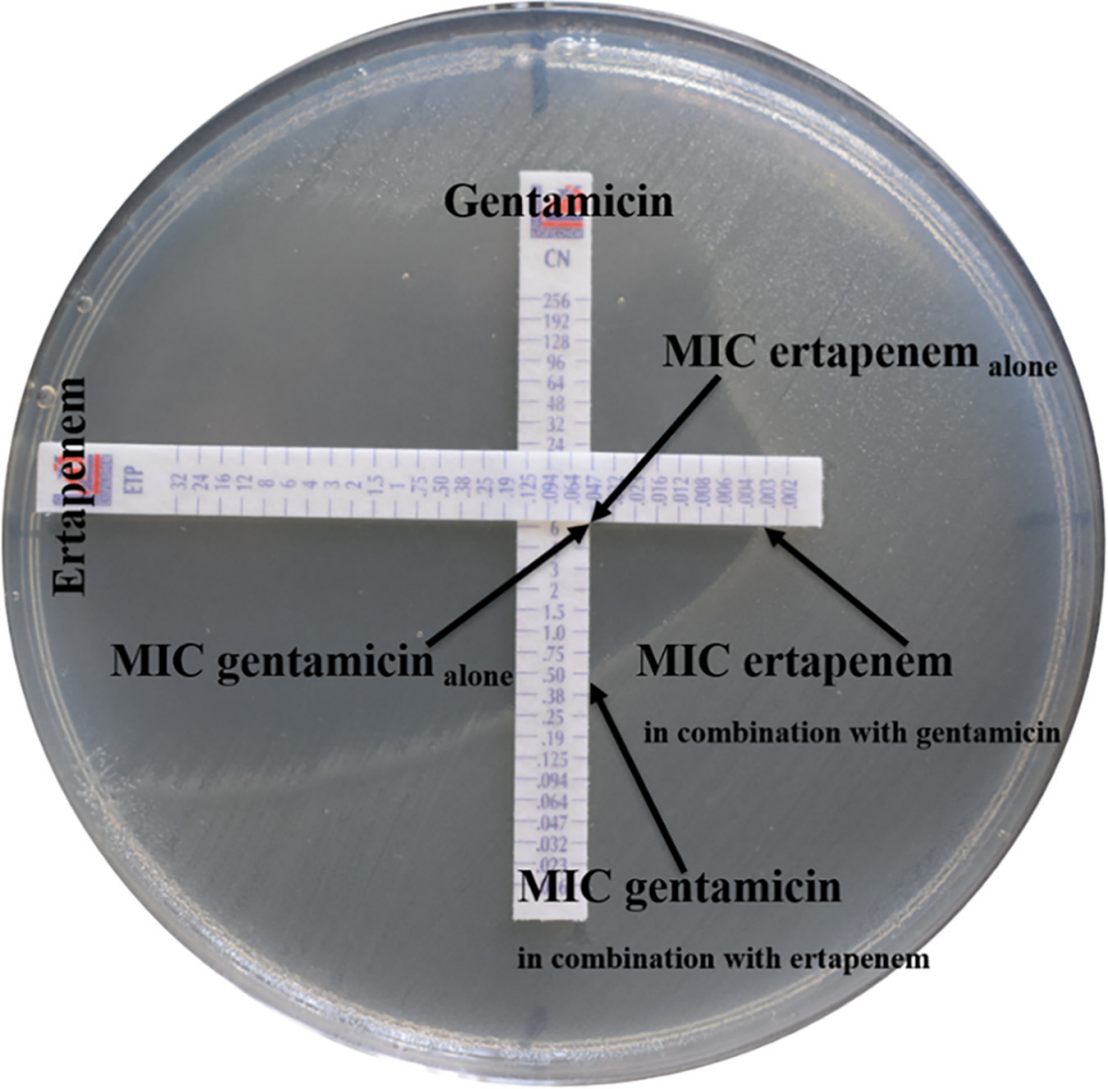
Photograph of strip placement for Etest synergy method. As an example, the E test strips of gentamicin and ertapenem were placed on the agar plates in a cross formation, with a 90° angle at the intersection between the scales at their respective MICs.

Of 407 clinical isolates of N. gonorrhoeae, 97 MDR isolates (determined below) were selected for antimicrobial combination testing (synergy/antagonism) according to MDR criteria. WHO P was used as a quality control strain. Dual antimicrobial testing was performed to evaluate the efficacy of gentamicin in combination with either ceftriaxone, ertapenem, or azithromycin using the Etest method, described previously (23) and demonstrated here, as an example, using gentamicin and ertapenem (Fig. 2). Briefly, MICs for individual antimicrobials (MIC gentamicin alone and MIC ertapenem alone) against Neisseria gonorrhoeae isolates were determined using Etest strips (Liofilchem, Italy); *in vitro* activity of each combination was determined by placing Etest strips of the two antimicrobials on the agar plates at a 90° angle, with intersections at the points of their individual MICs. Agar plates were inverted during incubation at 36°C in 5% CO_2_ for 16 to 18 h, and the MIC of each antimicrobial in the combination (MIC gentamicin in combination with ertapenem and MIC ertapenem in combination with gentamicin) was read. To determine whether each antimicrobial combination resulted in a synergistic, indifferent, or antagonistic effect, the fractional inhibitory concentration index (FICI) was calculated using the following formula: FICI = (MIC gentamicin in combination with ertapenem/MIC gentamicin alone) + (MIC ertapenem in combination with gentamicin/MIC ertapenem alone) (23). FICI values were interpreted using the following criteria: synergy, ≤0.5; indifference, FICI of >0.5 to ≤4.0; and antagonism, FICI of >4.0 (23).

### Statistical analysis.

The chi-square test was used to compare gentamicin susceptibility trend data and the categorical assignments of gentamicin susceptibility by the agar dilution or Etest method. Mean values of MICs and FICIs were calculated as geometric means (GM). The statistical significance of the difference between the MIC of each of the three antimicrobials tested alone and in combination with gentamicin was determined using the nonparametric Mann-Whitney U test. *P* values less than 0.05 were considered statistically significant.
