# Supplementary material for: In Vitro Efficacy of Gentamicin Alone and in Combination with Ceftriaxone, Ertapenem, and Azithromycin against Multidrug-Resistant Neisseria gonorrhoeae
Source: Microbiol Spectr. 2021 Oct 20;9(2):e00181-21. doi: 10.1128/Spectrum.00181-21 (PMC8528116; doi:10.1128/Spectrum.00181-21)
Supplement: SUPPLEMENTAL FILE 1 — Supplemental material. Download SPECTRUM00181-21_Supp_1_seq2.pdf, PDF file, 0.6 MB [file spectrum00181-21_supp_1_seq2.pdf]

Supplemental Tables

Supplemental Table 1. Phenotypic characteristics of 97 MDR *Neisseria gonorrhoeae* isolates selected for synergy test

Type		Number	Category I antibiotics		Category II antibiotics		
			CRO	CFM	AZM	CIP	PEN
MDR-NG	A	1	DS	DS	R	R	R
	B	2	DS	S	R	R	R
	C	23	S	DS	S	R	R
	D	28	DS	DS	S	R	R
	E	42	DS	S	S	R	R
	F	1	S	DS	R	R	R

Abbreviations: MDR-NG, multidrug-resistant *Neisseria gonorrhoeae*; CRO, ceftriaxone; CFM, cefixime; SPT, spectinomycin; AZM, azithromycin; CIP, ciprofloxacin; PEN, penicillin; DS, decreased susceptibility; S, susceptible; R, resistant.

Supplemental Table 2. Comparison of gentamicin MICs determined by agar dilution method and Etest for 97 MDR strains of *N. gonorrhoeae*

Etest MIC (mg/L)	Agar dilution MIC (mg/L)			
	2	4	8	16
2	2[a]	1		
3	1	3	3	
4		2	16	1
6			25	2
8			23	14
10				1
12				3

[a]The number in each cell represents the number of isolates that display the indicated MICs.

Supplemental Table 3. Comparisons with synergy testing from published studies using gentamicin in combination with ertapenem, ceftriaxone and azithromycin

Combination	Country	Year[a]	Isolates	Method	S (%)	A (%)	FICI	Interpretations	Reference
GEN+CRO	India	2013-2017	95 isolates including 79 MDR and one XDR isolate	Etest	14.7	6.3	0.977	Indifference	（1）
	US	2007-2012	32 isolates displaying varying cefixime MICs	Etest	0	0	1.25	Indifference	（2）
	Canada	-	9 WHO reference strains	Etest	0	0	1.2	Indifference	（3）
	China	2016-2017	MDR	Etest	16.5	0	0.747	Indifference	This study

GEN+ETP	India	2013-2017	95 isolates including 79 MDR and one XDR isolate	Etest	31.6	0	0.603	Indifference	（1）
	China	2016-2017	MDR	Etest	27.8	0	0.662	Indifference	This study
GEN+AZM	India	2013-2017	95 isolates including 79 MDR and one XDR isolate	Etest	11.6	6.3	1.257	Indifference	（1）
	India	2013-2017	70 isolates displaying varying ceftriaxone MICs	Etest	22.9	0	0.952	Indifference	（4）
	UK	2007 - 2010	64 isolates displaying varying cefixime MICs	AD	0	0	1.7	Indifference	（5）
	Japan	2011	25 isolates displaying varying ceftriaxone MICs	CB	0	0	0.83	Indifference	（6）
	China	2016-2017	MDR	Etest	8.2	0	1.021	Indifference	This study

Abbreviations: GEN, gentamicin; CRO, ceftriaxone; ETP, ertapenem; AZM, azithromycin; S, synergy; A, antagonism; MDR, multidrug-resistant; XDR, extensively drug resistant; MIC, minimum inhibitory concentration; AD, agar dilution; CB, checkerboard; FICI, fractional inhibitory concentration.

[a]Year of strain collection.

REFERENCES

1. Singh, V., M. Bala, A. Bhargava, M. Kakran, and R. Bhatnagar**.** 2018. In vitro efficacy of 21 dual antimicrobial combinations comprising novel and currently recommended combinations for treatment of drug resistant gonorrhoea in future era. PLoS One 13**:**e0193678.
2. Barbee, L. A., O. O. Soge, K. K. Holmes, and M. R. Golden**.** 2014. In vitro synergy testing of novel antimicrobial combination therapies against *Neisseria gonorrhoeae*. J Antimicrob Chemother 69**:**1572-1578.
3. Bharat, A., I. Martin, G. G. Zhanel, and M. R. Mulvey**.** 2016. In vitro potency and combination testing of antimicrobial agents against *Neisseria gonorrhoeae*. J Infect Chemother 22**:**194-7.
4. Sood, S., S. K. Agarwal, R. Singh, S. Gupta, and V. K. Sharma**.** 2019. In vitro assessment of gentamicin and azithromycin-based combination therapy against *Neisseria gonorrhoeae* isolates in India. J Med Microbiol 68**:**555-559.
5. Pereira, R., M. J. Cole, and C. A. Ison**.** 2013. Combination therapy for gonorrhoea: in vitro synergy testing. J Antimicrob Chemother 68**:**640-643.
6. Furuya, R., Y. Koga, S. Irie, M. Tanaka, F. Ikeda, A. Kanayama, and I. Kobayashi**.** 2013. In vitro activities of antimicrobial combinations against clinical isolates of *Neisseria gonorrhoeae*. J Infect Chemother 19**:**1218-1220.
